# Phylogenetic Relationships of Palaearctic *Formica* Species (Hymenoptera, Formicidae) Based on Mitochondrial Cytochrome *b* Sequences

**DOI:** 10.1371/journal.pone.0041697

**Published:** 2012-07-23

**Authors:** Anna V. Goropashnaya, Vadim B. Fedorov, Bernhard Seifert, Pekka Pamilo

**Affiliations:** 1 Department of Ecology and Genetics, EBC, Uppsala University, Uppsala, Sweden; 2 Institute of Arctic Biology, University of Alaska, Fairbanks, Alaska, United States of America; 3 Senckenberg Museum of Natural History, Görlitz, Germany; 4 Department of Biosciences, University of Helsinki, Helsinki, Finland; Université Paris 13, France

## Abstract

Ants of genus *Formica* demonstrate variation in social organization and represent model species for ecological, behavioral, evolutionary studies and testing theoretical implications of the kin selection theory. Subgeneric division of the *Formica* ants based on morphology has been questioned and remained unclear after an allozyme study on genetic differentiation between 13 species representing all subgenera was conducted. In the present study, the phylogenetic relationships within the genus were examined using mitochondrial DNA sequences of the cytochrome *b* and a part of the NADH dehydrogenase subunit 6. All 23 *Formica* species sampled in the Palaearctic clustered according to the subgeneric affiliation except *F. uralensis* that formed a separate phylogenetic group. Unlike *Coptoformica* and *Formica* s. str., the subgenus *Serviformica* did not form a tight cluster but more likely consisted of a few small clades. The genetic distances between the subgenera were around 10%, implying approximate divergence time of 5 Myr if we used the conventional insect divergence rate of 2% per Myr. Within-subgenus divergence estimates were 6.69% in *Serviformica*, 3.61% in *Coptoformica*, 1.18% in *Formica* s. str., which supported our previous results on relatively rapid speciation in the latter subgenus. The phylogeny inferred from DNA sequences provides a necessary framework against which the evolution of social traits can be compared. We discuss implications of inferred phylogeny for the evolution of social traits.

## Introduction

Large scale molecular studies have recently focused on the phylogeny of ant subfamilies [1], [2]. Based on these studies, diversification of ants started in early Cretaceous 115–135 Mya, and the age of the subfamily Formicinae is about 80 Mya. One of the internal dating points used by Brady et al. [1] was 44.1 Mya for the fossils of the genus *Formica* found in Eocene amber [3], [4]. *Formica* ants represent a large group of soil insects that occur mainly in the Holarctic. The genus has currently 176 recognized species, a bigger part of which are distributed in the Nearctic and a smaller part (63 species) in the Palaearctic ([5], World Catalogue of Ants, www.antweb.org, 27 September 2011). Even though the genus is well studied, these numbers are still changing (e.g. [6], [7]). Many species are widespread and abundant, and they play an important role in ecosystems being active predators, tending aphids and improving soil composition. *Formica* species also demonstrate a great diversity of complex behavior and social organization. The subgenus *Raptiformica* includes slave-making species, and the subgenera *Formica* s. str. and *Coptoformica* use temporary parasitism as a mode of founding new colonies, while the species of the subgenus *Serviformica* are used as slaves. The organization of colonies ranges from simple monogynous societies to huge supercolonies [8], [9], [10].

It is often concluded that facultative behavioral responses as well as traits connected to some socially important features have evolved independently several times across lineages (e.g. [11], [12]). On the contrary, the army ant syndrome or the complex of specific behavioral and reproductive traits has been shown to have evolved once rather than independently in the Old and New Worlds as thought previously [13]. Variation of social characteristics has made *Formica* ants useful for studying the evolution of social organization and for testing different theoretical implications of the kin selection theory (e.g. [14]). For example, Boomsma and Sundström [15] made a comparative phylogenetic study on the evolution of polyandry, looking for the correlation between the frequency of multiple mating and the paternity skew in seven *Formica* species in which single mating is still common. As no phylogenetic data were available, they used ten different phylogenetic hypotheses for testing the statistical significance of the observed relationship. Similarly, Helanterä and Sundström [16] studied whether colony size or colony kin structure had affected the evolution of worker egg-laying, and they corrected the phylogenetic dependencies by using our preliminary results. The phylogeny (as inferred e.g. from DNA sequences) provides the necessary background against which the evolution of the other traits can be compared.

Most taxonomists have distinguished four subgenera in the European *Formica* species (e.g. [3], [17], [18]), namely *Raptiformica*, *Coptoformica*, *Serviformica*, and *Formica* s. str. In addition, the only described species of the subgenus *Iberoformica* has a restricted distribution in the Iberian Peninsula [19]. Distinctions in morphological characters used for taxonomy of the genus often tend to be clear in local faunas but vague and imprecise when closely related species are studied on a large geographical scale. The previous allozyme study on 13 *Formica* species of four European subgenera [20] agreed with the subgeneric division based on morphological and behavioral characters with some exceptions. One of the exceptions was the topological position of *F. uralensis* (subgenus *Formica* s. str.) that was associated with *Serviformica*. According to Dlussky [3], *F. uralensis* belongs to *Serviformica* on the basis of the morphological similarities of the males. These characters were considered by him evolutionary conservative, while similarities in the worker morphology and nest constructing behavior of *F. uralensis* and *Formica* s. str. were believed to be secondary (convergent) traits. Due to a low level of allozyme variation in *Formica* ants, the resolution of the relationships of the species was poor with these data and the phylogeny of the genus remained unclear [20].

The present study aims to explore the phylogenetic relationships of the Palaearctic subgenera within genus *Formica*. One aim is to provide phylogenetic information which can be used in comparative studies such as those by Boomsma and Sundström [15] and by Helanterä and Sundström [16]. The other aim is to compare the divergence among species within and between the subgenera. The motivation for this is that the species in the *Formica rufa* group (*Formica* s. str.) form a morphologically variable and poorly differentiated group of species which hybridize (e.g. [21], [22], [23]). We have used mitochondrial DNA (mtDNA) to examine the *Formica rufa* group [21] and shown that these species form a monophyletic group of closely related species. Our results generally supported the division of this group into distinct species suggested on the morphological basis. Yet the species are geographically widely distributed and show, even within a single species, a wide variation of social structures from monogynous societies to large supercolonies which resemble those in invasive ants (e.g. [9], [10], [21]). The results from the *F. rufa* group suggest recent radiation and we want to compare this pattern with that in the other subgenera.

## Materials and Methods

### Sampling and molecular techniques

We examined 35 individuals of 24 *Formica* species representing the four subgenera (Table 1). As an outgroup, we used five individuals of *Polyergus rufescens* from two locations in Germany because *Polyergus* has been suggested to be the sister group of *Formica* [24]. No specific permits were required for the described field collections. All locations were not privately-owned or protected in any way. The field studies did not involve endangered or protected species. All samples were stored in 70% ethanol until DNA extraction. Total genomic DNA was extracted from only the head and mesosoma of single individuals with the DNeasy Tissue Kit (QIAGEN Inc.).

**Table 1:** List of *Formica* species used in the study, their subgeneric groupings and sampling localities.

Species and Groupings	Locality	Accession Number
Subgenus *Formica* s. str.		
** F. truncorum**	Sweden	AY488789
** F. frontalis**	Spain	AY488790
** F. pratensis**	Finland	AY584199
** F. lugubris**	Switzerland	AY573885
** F. paralugubris**	Switzerland	AY488767
** F. aquilonia**	Sweden	AY488780
** F. polyctena**	Urals, Russia	AY488762
** F. rufa**	Belgium	AY517505
Species *F. uralensis*		
* F. uralensis*-I	Ulaanbaatar, Mongolia	JX170881
** F. uralensis** ***-II***	Germany	JX170879
** F. uralensis** ***-III***	Finland	JX170878
** F. uralensis** ***-IV***	Urals, Russia	JX170880
Subgenus *Coptoformica*		
** F. exsecta** ***-I***	Germany	JX170867
* F. exsecta*-II	Tibet, China	JX170868
** F. foreli** ***-I***	Öland, Sweden	JX170873
** F. foreli** ***-II***	Öland, Sweden	JX170873
** F. pressilabris** ***-I***	Urals, Russia	JX170871
** F. pressilabris** ***-II***	Urals, Russia	JX170872
** F. pisarskii**	Eastern Siberia, Russia	JX170876
** F. forsslundi**	Ullanbaatar, Mongolia	JX170877
** F. manchu** ***-I***	Eastern Siberia, Russia	JX170874
** F. manchu** ***-II***	Eastern Siberia, Russia	JX170875
Subgenus *Raptiformica*		
** F. sanguinea** ***-I***	Sweden	JX170891
** F. sanguinea** ***-II***	Leon, Spain	JX170890
** F. sanguinea** ***-III***	Eastern Siberia, Russia	JX170892
** F. sanguinea** ***-IV***	Ulaanbaatar, Mongolia	JX170892
** F. sanguinea** ***-V***	Altai, Russia	JX170892
Subgenus *Serviformica*		
** F. lemani**	Eastern Siberia, Russia	JX170882
** F. fusca**	Sweden	JX170888
** F. selysi**	Switzerland	JX170883
** F. cinerea**	Sweden	JX170884
** F. cunicularia**	Western Siberia, Russia	JX170885
** F. rufibarbis**	Sweden	JX170889
* F. picea*	Sweden	JX170886
* F. candida*	Kyrgyzstan	JX170887

GenBank accession numbers of *Formica* sequences are given.

A 1.5 kb mtDNA fragment including the cytochrome *b* gene (cyt *b*) was amplified and sequenced with the following primers: Cytb-Fe-F [25], CB1, CB2, CB3, tR^S^ [26], CB-11059, CB-11178, and CB-11449 designed by using of the Oligo Primer Analysis Software v. 6.45 [27]. Polymerase chain reaction (PCR) was carried out in 25 μL volumes containing 1×PCR buffer, 2.0 mM MgCl_2_, 0.4 μg/μL BSA, 0.2 mM dNTPs (MBI Fermentas), 0.4 μM of each primer and 2.0 U Taq polymerase (Fermentas). A program for the amplification in a thermal cycler was used as follows: 3 min at 94°C, 35 cycles of 30 sec at 92°C, 30 sec at 45°C, 1–2 min at 68°C, and 10 min at 72°C.

PCR products were cleaned with the QIAquick Gel Extraction Kit (QIAGEN Inc.) and then sequenced on an Applied Biosystems 3100 automated DNA sequencer using the ABI Prism Dye Terminator Cycle Sequencing Ready Reaction Kit (Applied Biosystems). In total, more than 1500 base pairs were obtained in 35 *Formica* individuals. Due to many substitutions in the intergenic regions in the sequences and ambiguities in alignment we included only the genes in the analysis: 292 bp from the NADH dehydrogenase subunit 6 (ND6), 1125 bp from cyt *b* and 24 bp from the transfer RNA with a UCN anticodon for serine (tRNA^Ser^), 1441 bp in total. For the analysis with the outgroup, we used only the partial ND6 and cyt *b* sequences, 1416 bp in total. All new data has been deposited in GenBank (Table 1, *Polyergus* accession numbers JX170869, JX170870).

### Data analysis

The simplest model of nucleotide substitution with the best fit to our data was selected on the basis of minimal value of the Akaike information criterion (AIC) that finds balance between goodness-of-fit and complexity of model by using the ModelGenerator software [28]. The transversion model (TVM) with correction for rate heterogeneity among nucleotide sites and empirical base frequencies, proportion of invariable sites (0.39), and alpha parameter of gamma distribution (0.39) was selected for the data set without an outgroup (1441 bp). The simplest model with the best fit to the data set with the outgroup *Polyergus rufescens* (1416 bp), was TVM with correction for rate heterogeneity among nucleotide sites and empirical base frequencies, proportion of invariable sites (0.40), and alpha parameter of gamma distribution (0.55). Maximum likelihood trees (ML) were constructed by using the nearest neighbor interchange tree search with MultiPhyl software [29]. Neighbor-joining (NJ) trees were constructed with PAUP* version 4.0b10 [30]. Sequence variation and substitution pattern of the 1.5 kb mtDNA fragment were analyzed using the program MEGA v. 3.0 [31]. We compared the log likelihood scores of maximum likelihood trees constructed with and without a molecular clock assumption [32] to evaluate constancy in rate of the sequence evolution among lineages.

## Results and Discussion

### Sequence variation and phylogenetic relationships among subgenera

**Figure 1:**
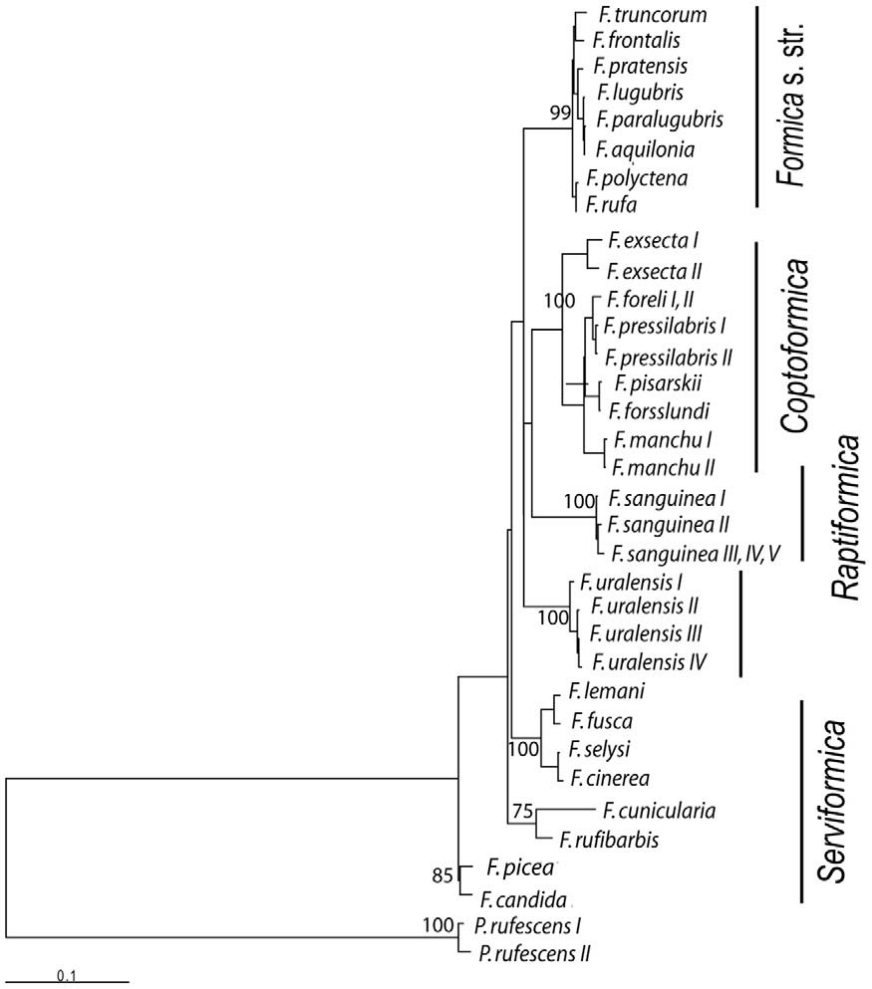
Maximum likelihood tree showing phylogenetic relationships among 32 mtDNA *Formica* haplotypes with the outgroup *Polyergus rufescens*. Bootstrap percentages with values over 70 are shown for major nodes. Specimens refer to Table 1.

**Figure 2:**
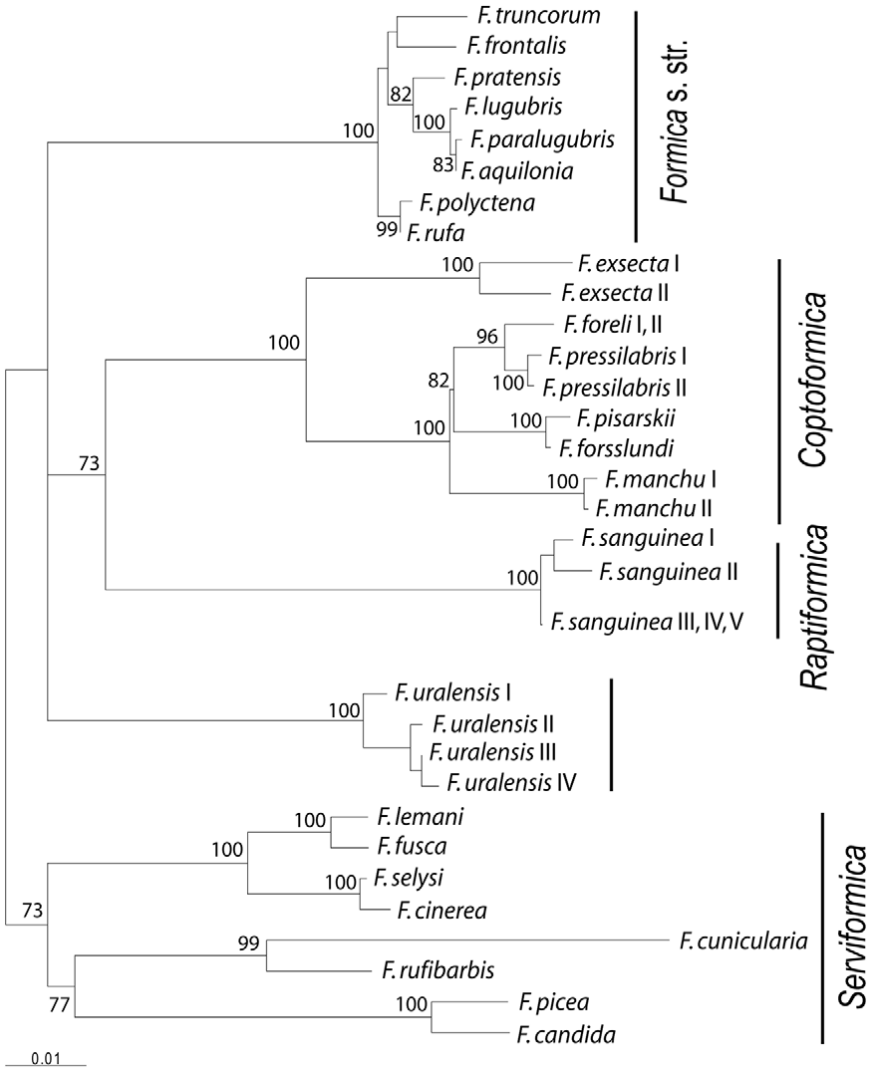
Maximum likelihood tree showing phylogenetic relationships among 32 mtDNA *Formica* haplotypes. Bootstrap percentages with values over 70 are shown for nodes. Specimens refer to Table 1.

Over the entire 1441-bp region examined in the *Formica* species (excluding *Polyergus*), 327 nucleotide positions were variable with 266 parsimony informative polymorphic sites. The *Polyergus* sequence used as the outgroup was too distant for reliable rooting of the tree as the synonymous nucleotide positions were saturated. The TVM+I+G distances between *Polyergus* and the *Formica* species were within a range from 0.50 to 0.65 and exceeded more than fourfold the distances among the *Formica* species (0.00–0.15) (Table 2). The use of non-synonymous substitutions alone did not resolve all the groupings within the genus *Formica*. The ML analyses based on all nucleotide positions with and without the outgroup produced similar trees. The tree including the outgroup showed the species pair *F. candida* and *F. picea* as basal to the rest of the species and *Serviformica* appeared paraphyletic. The species *F. candida* and *F. picea* are closely related and their taxonomical position was clarified only recently [33]. The tree without the outgroup separated *Serviformica* from the other species with only a moderate bootstrap support of 73% (Figures 1, 2). Topology of the NJ tree (not shown) without outgroup rooting was similar to the ML tree. The comparison of the likelihood scores of the ML trees constructed with and without the molecular clock assumption showed that the sequences have evolved at roughly constant rates (P = 0.15). Therefore, variation in mtDNA sequences is suitable for approximate estimation of relative divergence times.

**Table 2:** Genetic divergence estimates within and between the phylogenetic groups in *Formica* (%): average distances within the groups (the diagonal, in bold), mean uncorrected (below the diagonal) and net (above the diagonal) distances between the groups.

	1. *Formica* s. str.	2. *Copto-formica*	3. *Rapti-formica*	4. *F. uralensis*	5. *Servi-formica*
1	**1.18±0.21**	7.73±1.14	9.58±1.55	8.24±1.20	4.72±0.75
2	10.13±1.25	**3.61±0.39**	7.93±1.21	8.98±1.42	5.39±0.88
3	10.45±1.59	10.02±1.32	**0.57±0.18**	8.58±1.27	7.41±1.19
4	9.17±1.24	11.13±1.54	9.21±1.30	**0.68±0.18**	6.01±0.93
5	8.65±0.95	10.54±1.13	11.04±1.37	9.70±1.14	**6.69±0.75**

The subgenera *Formica* s. str. and *Coptoformica* were represented by several species and formed monophyletic clades respectively. *Serviformica* formed also a group of its own in an unrooted tree, but we could not reliably infer its monophyly because of problems with the root. The *Serviformica* species which have been studied have a haploid chromosome number n = 27, except *F. picea* which has n = 26 like all other *Formica* subgenera [34], [35]. Two species in our unrooted tree fell outside these three subgenera, namely *F. sanguinea* (subgenus *Raptiformica*) and *F. uralensis*. The systematic position of *F. uralensis* has been controversial in the past (e.g. [17]), and Dlussky [3] concluded that its male genitalia are most similar to those of *Serviformica*. We consider this a weak argument as the variation of male genitalia is poorly studied throughout the genus and unknown in many species. Furthermore, the haploid chromosome number (n = 26) departs from that of most *Serviformica* species [34]. Separate position of *F. uralensis* in the mtDNA phylogenetic tree agreed with the results from an earlier allozyme study [20] showing that this species represents a separate phylogenetic lineage and could be placed to a subgenus of its own.

Rooting of the phylogenetic tree was problematic as the outgroup sequence was too distant and the differences separating the subgenera were small. An earlier study by Hasegawa et al. [24] based on COI sequences included five *Formica* species from three subgenera and had *Polyergus* as the nearest outgroup. The branching order of the *Formica* species could not be solved with great confidence, but the unweighted maximum parsimony tree suggested *Serviformica* (represented by *F. fusca* and *F. cunicularia* in that study) as a basal subgenus with *Coptoformica* and *Formica* s. str. clustering together. This pattern agrees with the topology of the tree in Figures 1, 2. However, all the subgenera (*Formica* s. str., *Coptoformica*, *Raptiformica*, *Serviformica*, and the species *F. uralensis*) demonstrated a bush-like branching pattern with a low bootstrap support for any cluster containing two or more subgenera.

All genetic distances between the subgenera were around 10% implying approximate divergence time of 5 Myr if we use the average divergence rate of 2.0% per Myr suggested for insect mtDNA [36]. The genetic distance between *Formica* and *Polyergus* (0.5–0.65) would then suggest that these two genera can have diverged during the Eocene if the divergence rate has been similar. This agrees with fossil record as several species classified to the genus *Formica* have been recorded from the Baltic amber [4]. Dlussky [4] further concluded that *Formica* from the Late Eocene amber constitute an archaic group and most of them are not similar to living congeners. The sequence divergences estimated by us suggested that the subgenera included in this study are young compared to the age of the whole *Formica* clade since separation of the sister genera. The genus has Holarctic distribution. There are true Nearctic *Raptiformica* species, but no true Nearctic *Coptoformica* (the *F. exsectoides* species group is clearly different from the Palaearcitc species) and no ants comparable to the mound-building *Formica* s. str. If interchange between North America and Eurasia reached a peak in the Middle Pliocene (4 Mya) [37], most of the diversification has happened during or after that period. Although migration between the continents occurred during the Pleistocene, when sea levels dropped significantly during glacial periods, making a dry land connection between Alaska and Siberia, it included the cold-adapted groups only, not the forms restricted to temperate climates like *Formica* ants.

### Divergence within the subgenera

The subgenus *Formica* s. str. formed a tight cluster of species and the internal topology was similar to that obtained in the previous study on the *F. rufa* group [21]. The only difference was that the *polyctena*/*rufa* clade appeared here as basal to the rest of the species instead of the *truncorum*/*frontalis* clade which is generally considered to be basal to the *F. rufa* group (*F. aquilonia, F. lugubris, F. polyctena, F. rufa, F. paralugubris*). The reason for this was most probably that the level of variation in this subgenus is low compared to the divergence from other subgenera and the resolution therefore poor. The small interspecific distances in this subgenus, particularly within the *F. rufa* group, are noteworthy. These interspecific distances were smaller than the sequence divergence observed within the species *F. exsecta* and similar to the intraspecific divergence in *F. pressilabris*, *F. manchu* and *F. uralensis*. These comparisons support previous findings [21] suggesting that the speciation rate has been high within the *Formica rufa* group of mound-building ants. This could be in part associated to the changes in the social organization when limited dispersal of females from polygynous societies can lead to strong differentiation between populations and possibly to speciation if male dispersal is also restricted [38], [39]. The *F. rufa* group species show different types of social organization having largely monogynous and monodomous colonies in some populations, whereas the species *F. aquilonia*, *F. paralugubris* and *F. polyctena* are always polygynous and tend to build large supercolonies ([9], [10], but see [40]). However, similar variation in social organization is known in other *Formica* species, e.g. in *F. exsecta* [38] and *F. cinerea* [41] without clear signs of accelerated speciation rate or increase in intraspecific genetic divergence. The relative importance of social organization and phylogeographic history in the speciation process within this group could not be estimated from the present data alone. Genetic studies have recently revealed new species within the *F. rufa* group, probably differentiated as a result of geographical isolation in the Alps [42], [43]. The known species have also been shown to hybridize as detected from transspecific capture of mtDNA [44] or nuclear introgression and phenotypic clustering [22], [23], while the sympatric populations generally form separate gene pools [43], [45].

According to the present results, at least two highly divergent lineages occurred in the subgenus *Coptoformica*. *Formica exsecta* was genetically most distant from the other species. Our previous study on *F. exsecta* included one sample from Tibet that proved highly divergent from the other samples of the species [46]. In the present phylogenetic tree this Tibetian sample (*F. exsecta*-II) clustered together with the other conspecific sample showing reciprocal monophyly of *F. exsecta* relative to the other *Coptoformica* species. It should be noted that the present data did not include *F. mesasiatica* [3], a morphologically defined sister species of *F. exsecta* that occurs only in Central Asia [18]. Our previous study on *F. exsecta* from the Palaearctic region included *F. mesasiatica* from Kyrgyzstan and placed its haplotypes within the *F. exsecta* clade leaving *F. exsecta* paraphyletic [46].

Unlike the subgenera *Coptoformica* and *Formica* s. str., the subgenus *Serviformica* clustered with only limited bootstrap support (73%) that included three small clades with large differences both between and within them: *cinerea*/*fusca/lemani/selysi*; *cunicularia*/*rufibarbis*; and *candida/picea*. Notably, this subgenus is the largest Palaearctic group within genus *Formica* ([5], [6], [7], www.antweb.org). Divergence among the *Serviformica* species far exceeded that within the other subgenera and was partly comparable with the subgeneric differences. Further studies with sampling of Nearctic species and subgenera are needed to shed more light to the history of the genus and the possible monophyly of *Serviformica*.

The shallow phylogeny of the *Formica* s. str. clade and the low divergence estimate (1.18%) within this subgenus supported our previous results that speciation in this subgenus, particularly among the species of the *F. rufa* group, was relatively fast [21]. Bernasconi et al. [43] have reported from the Swiss Alps a population which may represent yet another species of the *F. rufa* group, and Kulmuni et al. [23] showed that some type of incompatibility factors keep populations with apparent hybrid background viable but separate. On the contrary, the *Serviformica* subgenus was highly diverged (within-subgenus divergence estimate was 6.69%) with possible subdivisions, even though the phylogeny is incomplete as only eight species were studied. *Formica uralensis* showed low genetic divergence despite its clear distinction from the other phylogenetic groups. Sequencing of the same mtDNA fragment from 11 samples of this species throughout its European distribution range revealed the nucleotide diversity as low as 0.19% [47]. Similar and even lower diversity estimates have been detected in the *F. rufa* group species [21]. It is possible that *F. uralensis* represents the only species that survived in this lineage and underwent reduction in historical effective size during the last glacials like the *F. rufa* group species

### Character evolution

All the subgenera (including *F. uralensis*) except *Serviformica* share some important social characteristics. They build mound nests with plant materials (even though the mound formation is not always clear in *F. sanguinea* and *F. truncorum*) and it is generally considered that they have lost the ability of independent single-queen colony foundation. When colonizing a new locality, they found colonies either by temporary social parasitism or by colony fission, or they are dulotic (*F. sanguinea*) and use slaves of another species. The *Serviformica* species do not build mounds with plant materials, they are capable for single-queen colony foundation, they are used as hosts during the socially parasitic colony founding by *F. sanguinea*, *F. uralensis*, and all species of *Coptoformica* and *Formica* s. str., and they also serve as slaves in *F. sanguinea* colonies. The branching order given in Figures 1, 2 suggests that the mound-building behavior and parasitism could have evolved only once before the other subgenera separated from each other. A molecular phylogeny of closely related Formicine species by Hasegawa et al. [24] showed that slave-making behavior has evolved independently in two closely related genera, *Polyergus* and *Rossomyrmex*. Both genera are more closely related to the genera they parasitize (*Formica* and *Proformica*, respectively) than to each other. Moreover, other phylogenetic studies on ants have indicated that social parasitism has originated independently several times in *Myrmica* [48]. Even though the phylogenetic clustering (Figures 1, 2) indicates the possibility that the nesting behavior including mound building with plant particles and temporary social parasitism may have evolved once in the ancestral lineage, the short internal branch, uncertainty of rooting, and the high frequency of interspecific parasitism in ants in general [49], make such a conclusion premature.

In addition to inferring the origin of qualitatively defined traits (such as parasitism), a phylogenetic tree and phylogenetic contrasts have been used to evaluate the evolution of quantitative traits. Using this approach, Boomsma and Sundström [15] presented the data from seven *Formica* species showing a negative association between the paternity skew and the frequency of double mating. In other words, the more frequent double mating is, the more evenly sperm is used by females. The analysis taking into account putative phylogenetic relationships of the species showed that the result was robust. It is therefore likely that the general conclusion holds even when none of the ten hypothetical trees used by Boomsma and Sundström [15] agrees with the result obtained here. Nevertheless, the present results provide a background against which such comparative studies can be made, as demonstrated by the comparative study of worker reproduction by Helanterä and Sundström [16].
